# Metallic one-dimensional heterostructure for gas molecule sensing

**DOI:** 10.1038/s41598-020-79921-8

**Published:** 2021-01-11

**Authors:** Prabal Dev Bhuyan, Sanjeev K. Gupta, Rajeev Ahuja, P. N. Gajjar

**Affiliations:** 1 Computational Materials and Nanoscience Group, Department of Physics and Electronics, St. Xavier’s College, Ahmedabad, 380009 India; 2 Department of Physics, University School of Sciences, Gujarat University, Ahmedabad, 380009 India; 3 Condensed Matter Theory Group, Department of Physics and Astronomy, Uppsala University, Box 516, 75120 Uppsala, Sweden; 4 Applied Materials Physics, Department of Materials and Engineering, Royal Institute of Technology (KTH), 100 44 Stockholm, Sweden

**Keywords:** Nanoscale devices, Materials for devices

## Abstract

We have investigated a new metallic core–shell nanowire (NW) geometry of that could be obtained experimentally, that is silicon (Si) and germanium (Ge) NWs with cores constituted by group-10 elements palladium (Pd) and platinum (Pt). These NWs are optimized with two different diameters of 1.5 Å and 2.5 Å. The nanowires having diameter of 1.5 Å show semi-metallic nature with GGA-PBE calculation and metallic nature while spin orbit interaction (SOC) is included. The quantum conductance of the NWs increases with the diameter of the nanowire. We have investigated current–voltage (IV) characteristics for the considered NWs. It has been found that current values in accordance with applied voltage show strong dependence on the diameter of the NWs. The optical study of the NWs shows that absorption co-efficient peak moves to lower energies; due to quantum confinement effect. Furthermore, we have extensively studied optical response of Pd and Pt based core–shell NWs in O_2_ and CO_2_ environment. Our study on Si and Ge based metallic core/shell NW show a comprehensive picture as possible electron connector in future nano-electronic devices as well as nano gas detector for detecting O_2_ gas.

## Introduction

The rising environmental concerns and the effect of pollution on health call lead to worldwide problem. As a result gas sensing has become as field of continuous research to detect industrial and automobile emissions and for monitoring our environment and household safety. These concerns have led to recommendations on the development of high performance gas sensors with high sensitivity and selectivity^1,2^. The sensing sensitivity can be enhanced by increasing the contact interface between the gas molecules and sensing materials^3^. Therefore, one dimensional (1D) nanomaterials are considered to be promising material with high sensitivity and selectivity due to large surface to volume ratio and enormous reaction sites^4^.

In this regard, the 1D materials have synthesized and showed potential application as gas sensing material^4^. The change in conductivity of gold (Au) nanowire with the gas molecules shows the potential for gas sensor^4,5^. Beyond Au-NW, metal nanowires such as silver (Ag)^6^, palladium (Pd)^7,8^ have also been studied and developed as sensors. Silicon (Si) nanowire has also attracted much attention as for ultra-fast chemical sensing systems^9,10^. A. Miranda and coworkers have reported the change in band gap upon the adsorption various gas molecules on the surface of Si-NW attributes its application in the field of sensing^9^. In addition, it is observed that nanowires combined with metal nanoparticles, enhances the reacting properties of the materials due to their unique chemical and physical properties^11–13^. Beyond that, heterostructure core–shell nanowires are also studied and synthesized for detecting gas molecules. Dong Liu et al. have created lower power consumption gas sensor from Si–TiO_2_ core–shell NW and successfully demonstrated sensing performance for CH_4_ detection at room temperature^14^. Further, Daejong Yang et al. have fabricated flexible gas sensor based on ZnO/ZnS core − shell NW^3^.

However, silicon (Si) and germanium (Ge) nanowires with cores constituted by transition metal group-10 element Pd and Pt have not been exploited yet. Both Pd and Pt nanowires have been synthesized experimentally and gained a high interest^15,16^. Pd nanowire has shown applications in the field of sensing as hydrogen detector^8,17^. Similarly, it has been reported that Pt nanowire could find applications as electro-catalysts in proton exchange fuel cells, high performance hydrogen sensors etc.^18,19^. Therefore, we intend to explore the electronic and optical properties of the Si and Ge shell based Pd and Pt core nanowire systems for gas detection.

In this work, we have focused on Si or Ge based metallic Pd- and Pt-core/shell nanowires and studied their geometry and electronic properties. Diameter dependent I–V characteristics and optical properties are also investigated in order to provide a picture as comprehensive as possible. Furthermore, we have adsorbed O_2_ and CO_2_ gas molecules on the considered core–shell nanowires to study the response of the nanowires with gas molecules and highlighted the optical sensitivity to investigate the suitability of the nanowires as a gas sensor.

## Methodology

Our present calculations are based on density functional theory (DFT) methods using SIESTA package for electronic structure and transport properties^20^. Generalized gradient approximation (GGA) using Perdew–Burke–Ernzerhof (PBE) is adopted for the exchange and correlational functional^21^. We have used Troullier–Martins norm-conserving and relativistic pseudopotential to account for core electrons and double zeta polarized basis set to describe valence electrons^22^. The ground state nanowire geometries are relaxed with conjugate gradient (CG) algorithm. Convergence on the density matrix during a self-consistent cycle was set to 10^–6^ eV. We have considered sufficient vacuum of 20 Å in x and y-direction to avoid interaction between the neighboring nanowire images. The reciprocal space is sampled at 1 × 1 × 12 using Monkhorst Pack meshes. We include 30 K-points in the band structure along Γ to Z.

The current–voltage (IV) characteristics graph of the optimized structure have been calculated by using non-equilibrium Green’s function (NEGF) technique, with Keldysh formalism based on density functional theory^23^. A two probe system is designed to study the IV properties of the core–shell NW confined in the central scattering region (SR) contact with semi-infinite left electrode (LE) and right electrode (RE). We have considered both electrodes and SR are made of the same material. Our goal is to compute transmission function, which can be obtained from the Green's function of the system. The transmission function T(E) is given by^24^,

1 $$T(E) = Tr\left[ {G(E)\Gamma_{R} (E)G(E)\Gamma_{L} (E)} \right]$$

$\Gamma_{L} (E)$ and $\Gamma_{R} (E)$ are the broadening matrices. G(E) is the Green’s function. The Green’s function G(E) of the SR is calculated from the formula,

2 $$\left( {ES - H} \right)G(E) = I$$

where, S is the overlap matrix, H is the Hamiltonian and I is the identity matrix. The Hamiltonian is composed of as follows,

$$\left( {\begin{array}{*{20}c} {H_{L} + \sum_{L} } & {H_{LC} } & 0 \\ {H_{LC} } & {H_{C} } & {H_{RC} } \\ 0 & {H_{RC} } & {H_{R} + \sum_{R} } \\ \end{array} } \right)$$

Here, L, C and R denote the left electrode, central region and right electrode, respectively. $\sum_{L}$ and $\sum_{R}$ are the self-energies for the two electrodes.

From transmission function, the current through the contact region is calculated,

3 $$I\left( {V_{bias} } \right) = G_{o} \int\limits_{{\mu_{R} }}^{{\mu_{L} }} {T\left( {E,V_{bias} } \right)dE}$$

where, $G_{o} = 2{{e^{2} } \mathord{\left/ {\vphantom {{e^{2} } h}} \right. \kern-\nulldelimiterspace} h}$ is the unit of quantum conductance and $T\left( {E,V_{bias} } \right)$ is the transmission probability of the electron incident with an energy E through the device under the potential bias $V_{bias}$. The bias voltage between the two electrodes of different electrochemical potential $\mu_{L}$ and $\mu_{R}$ is given by $eV_{bias} = \mu_{L} - \mu_{R}$.

Further, we have investigated the optical properties of the NWs for the both diameters and analyzed the variation of the properties with gas adsorbed NWs for sensing application. We have considered only interband transition for the optical properties calculation. The frequency dependent complex dielectric function can be written as $\varepsilon = \varepsilon_{1} + i\varepsilon_{2}$. $\varepsilon_{1}$ is the real part of the dielectric function and is determined from the Kramers–Kronig (KK) relationship. $\varepsilon_{2}$ is the imaginary part of the function is obtained from the summation over electronic states. The $\varepsilon_{2}$ depicts the absorption behaviour of the material, which can be written as;

4 $$\alpha \left( \omega \right) = \sqrt 2 \omega \left[ {\left\{ {\varepsilon_{1}^{2} \left( \omega \right) + \varepsilon_{2}^{2} \left( \omega \right)} \right\}^{{{\raise0.7ex\hbox{$1$} \!\mathord{\left/ {\vphantom {1 2}}\right.\kern-\nulldelimiterspace} \!\lower0.7ex\hbox{$2$}}}} - \varepsilon_{1} \left( \omega \right)} \right]^{{{\raise0.7ex\hbox{$1$} \!\mathord{\left/ {\vphantom {1 2}}\right.\kern-\nulldelimiterspace} \!\lower0.7ex\hbox{$2$}}}}$$

## Results and discussions

### Structure and stability

**Figure 1:**
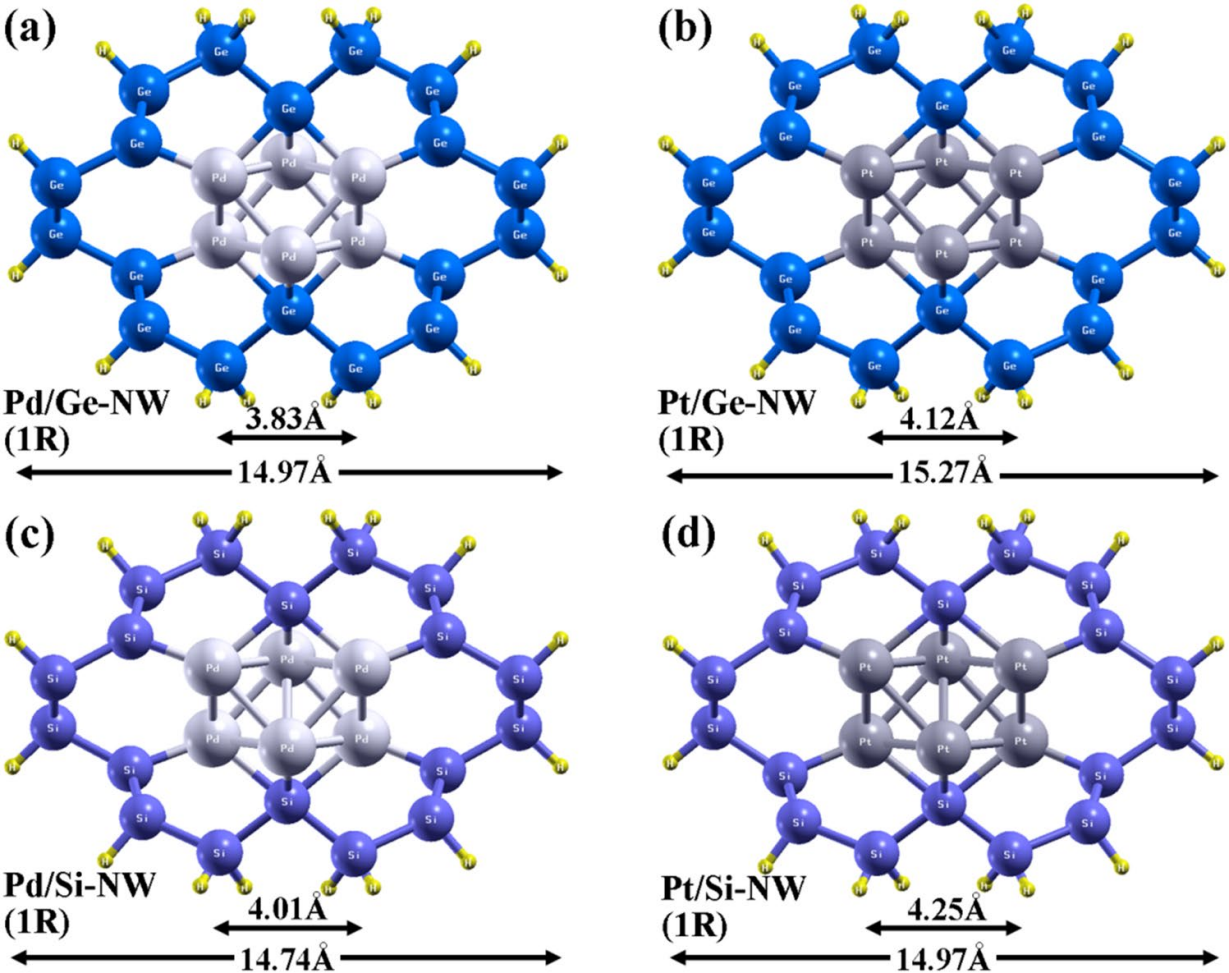
Front view of the fully optimized structures of (**a**) Pd/Ge, (**b**) Pt/Ge, (**c**) Pd/Si and (**d**) Pt/Si core–shell NWs. The core is wrapped by 1ML Ge or Si-shell. Hydrogen atoms are passivated on the surface of the NWs to prevent the dangling bonds.

We have considered two different cores; palladium (Pd) and platinum (Pt), which are wrapped by germanium (Ge) and silicon (Si) shell. Therefore, we have four different core–shell NW configurations: Pd_core_/Ge_shell_, Pt_core_/Ge_shell_, Pd_core_/Si_shell_ and Pt_core_/Si_shell_. The surface of the NWs is passivated by hydrogen atom to prevent dangling bond. The relaxed and optimized geometry of core–shell NWs are shown in Fig. 1. The core of the considered core–shell NWs is constituted with 6 atoms of Pd or Pt and the thickness of the Si or Ge shell is varied with nanowire size. Considering Pd/Ge NW, the core wrapped by one monolayer (1ML) of Ge shell has 40 atoms (6 Pd atoms, 18 Ge atoms and 16 H atoms) with a diameter of ~ 1.5 nm. The diameter increases to ~ 2.3 nm while the core is wrapped by 2MLs of Ge shell with 78 atoms (6 Pd atoms, 48 Ge atoms and 24 H atoms). The core with 1ML shell is denoted by 1R (Fig. 1a) and with 2ML shells is denoted by 2R (ESI, Figure S1a). The NWs are considered along the [110] direction, which is reportedly preferred growth direction in case of synthesized Si NW and also Ge/Si core–shell NW^25^. We have observed that the variation in diameter of the NWs is according to the radius of atoms also.

In order to comprehend the energetic stability of the morphology, we have calculated cohesive energy of the NWs from the following equation;

5 $$E_{coh} = \frac{{E_{T} - nE_{a} }}{n}$$

where, $E_{T}$ is the total energy of the core–shell NW, $E_{a}$ is the energy of a free atom and n is the total number of atoms in the nanowire. We have obtained negative cohesive energy for the core–shell nanowires (see in Table 1). The negative values of cohesive energy ($E_{coh}$) indicates the stability of the nanowires. It is observed that $E_{coh}$ increases with the diameter of the respective nanowire. The NWs shows cohesive energy in the range of − 3.6 ~ − 3.8 eV, while Pd and Pt core wrapped with Ge shell. The energy range decrease to − 4.1 ~ − 4.4 eV, while Pd and Pt core wrapped with Si shell. It is reported that pure Pd-NW having diameter of 2.57 Å shows cohesive energy of ~ − 2.4 eV and Pt-NW having diameter of 2.61 Å shows cohesive energy of ~ − 4.2eV^26^.

**Table 1:** Cohesive energy (E_coh_) and quantum conductance of the considered core–shell NWs.

Core–shell NW	Pd/Ge		Pt/Ge		Pd/Si		Pt/Si	
	1R	2R	1R	2R	1R	2R	1R	2R
E_coh_ (eV/atom)	− 3.68	− 3.80	− 3.66	− 3.79	− 4.16	− 4.43	− 4.14	− 4.42
Quantum conductance (G_o_)	2	12	2	10	2	10	2	14

### Electronic properties

**Figure 2:**
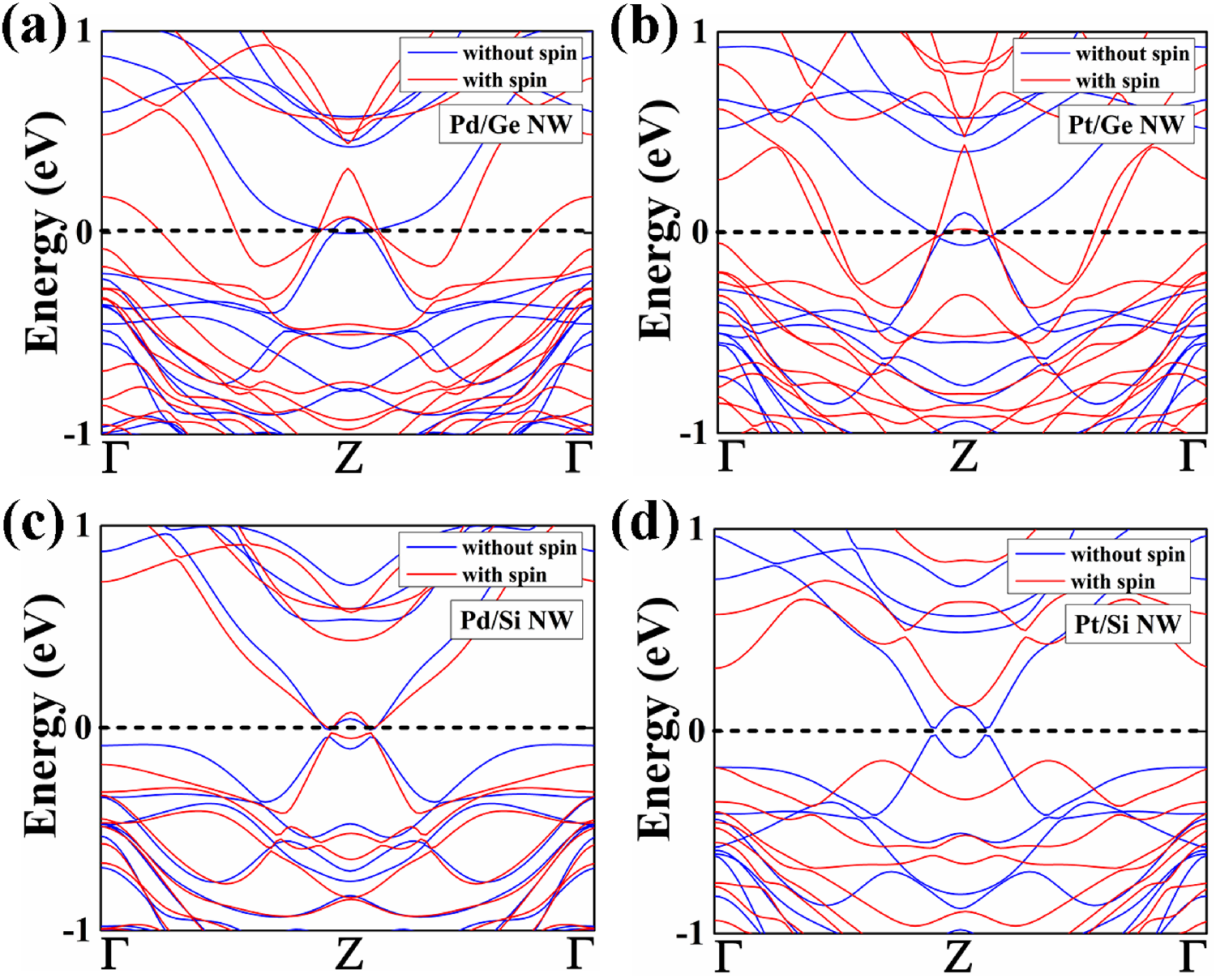
The electronic band structure without spin–orbit interaction and within spin–orbit interaction of (**a**) Pd/Ge, (**b**) Pt/Ge, (**c**) Pd/Si and (**d**) Pt/Si NWs having diameter of 1.4 ~ 1.5 nm (1R) is shown. The Fermi level is set at 0 eV.

**Figure 3:**
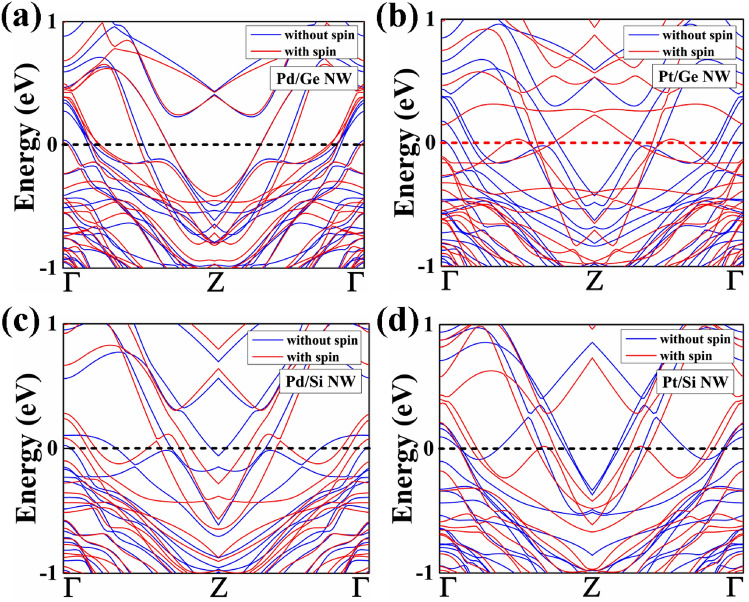
The electronic band structure without spin–orbit interaction and within spin–orbit interaction of (**a**) Pd/Ge, (**b**) Pt/Ge, (**c**) Pd/Si and (**d**) Pt/Si NWs having diameter of 2.2 ~ 2.3 nm (2R) is shown. The Fermi level is set at 0 eV.

After the structural optimization, we have focused on the electronic properties of these core–shell nanowires. We have studied both the with spin–orbit interaction and the without spin orbit interaction electronic band structure. The electronic band structures of 1R and 2R NWs are shown in Figs. 2 and 3, respectively.

The core–shell NW with 1ML (1R) shows semi-metallic behaviour without spin–orbit interaction calculation as a single Fermi level crossing near the zone boundary. The electronic band structure shows that 2G_o_ quantum conductance as the bands line touches the Fermi energy level at two points near the Z-point. However, Pd/Ge and Pt/Ge NW shows metallic behaviour with quantum conductance of 4G_0_, while spin–orbit interaction is included. Pd/Si NW shows simillar semimetallic behaviour from both the with spin–orbit interaction and the without spin–orbit interaction calculation. In case of Pt/Si NW, there is a band gap of 0.26 eV appears at the Fermi level (E_f_) which shows semiconducting behaviour using spin–orbit interaction. Furthermore, we have found that the increase in the diameter of core–shell NWs results in the transition of electronic properties. The Pd and Pt core wrapped by 2ML Ge and Si shell NWs show metallic nature, which means resistivity decreases with the diameter. The decreasing resistivity with increasing nanowire diameter is explained by Fuchs–Sondheimer (FS) theory. As the size of our considered core–shell nanowires are shorter than the mean free path of the electron, therefore, the surface scattering influences the resistance of a nanowire^27,28^ which leads to the decrease in resistivity. Pd/Ge and Pt/Si NW show conductance of 12G_o_ and 14G_o_, respectively from both the with and without spin orbit interaction. Though both the NWs shows same conductance value, but the change in electronic band structure is observed near the Fermi region. The other two nanowires Pt/Ge and Pd/Si NW shows variation in conductance for the spin orbit interactiona and without spin orbit interactiona calculation. Pt/GeNW shows conductance of 8G_o_ (10G_o_) under spin orbit interaction (without spin orbit interaction). Further, Pd/Si NW shows conductance of 6G_o_ (14G_o_) under spin orbit interaction (without spin orbit interaction). So, we have observed that as the diameter of the nanowires decreases, the band gap is increased. The increase of band gap is due to the quantum confinement effect.

To study the orbital contribution of the nanowires near the Fermi energy level, we have studied the partial density of states and are shown in ESI (Figure S2 and S3). The contribution of both core and shell is observed near the Fermi energy. In the case of 1R NWs, the core, Pd-4d and Pt-5d orbital electron are more dominant than the shell region on the valence band. However, at the conduction band, the contribution of the shell region; Ge-4p and Si-3p orbital is observed to be higher. The contribution of orbitals notably changes as the diameter of the NWs increases from 1 to 2R. It is observed that both the valance band and conduction band is largely contributed by the shell region. The Ge-4p and Si-3p are actively dominant near the Fermi energy level. Thus, the Si and Ge shells of different thicknesses lead to a tuning of the electronic structure of the Pd and Pt nanowires through a state of hybridization between the core and shell orbitals at the interface.

**Figure 4:**
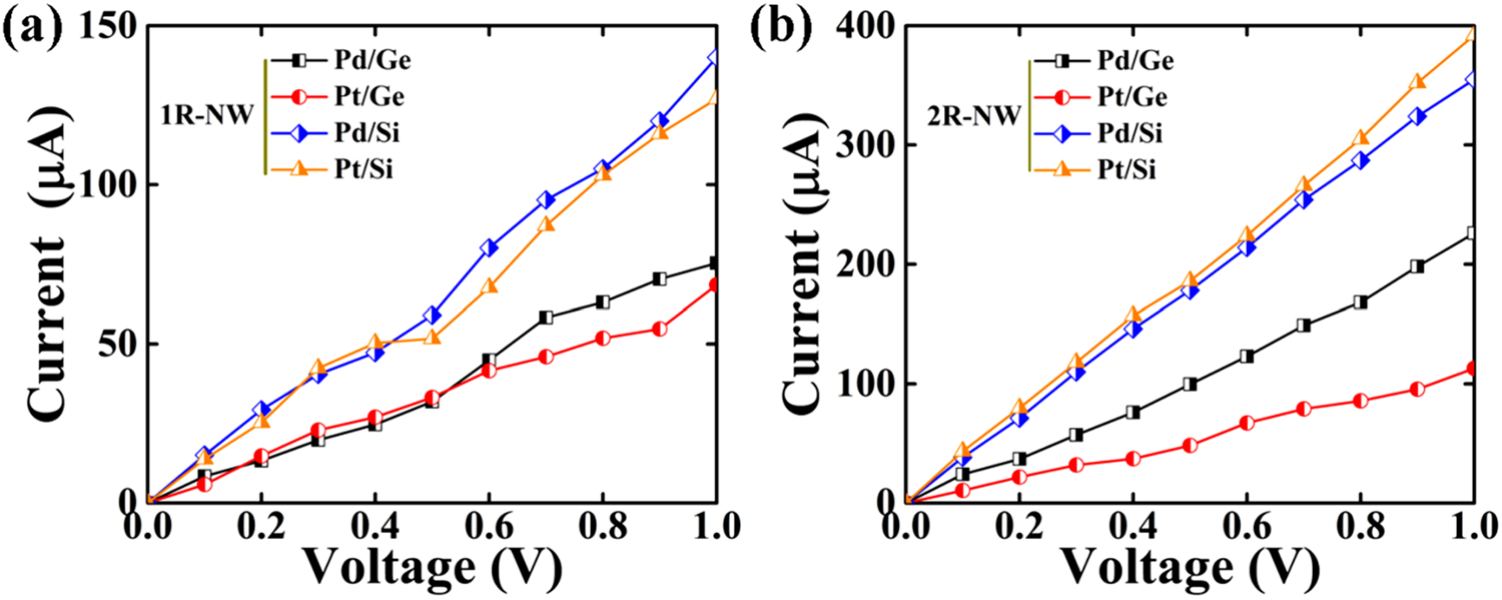
Current–Voltage (I–V) plot for (**a**) 1R and (**b**) 2R core–shell NWs for bias voltages from 0 to 1 V.

We have observed that quantum conductance of the core–shell NWs increases with increase in diameter. To support the electronic structure study, we have studied the I–V characteristics of the core–shell NWs using NEGF technique as discussed in methodology section. We have considered a two probe system of electrodes (LE and RE) and scattering region (SR). The length of the SR is 12 ~ 13 Å with three primitive unit cells and is sufficient enough to avoid an abrupt change in electronic structure. We have increased V_bias_ in the steps of 0.1 V and used converged density matrix of the previous step as the initial guess for the next step. By applying a bias voltage, the Fermi level of the left electrode shifts with respect to the Fermi level of the right electrode. While, the energy of the top of the valance band of the left electrode matches with the energy of the bottom of the conduction band of the right electrode, the current starts flowing through the system. The current as a function of the applied bias voltage (V_bias_) is present in Fig. 4.

We have observed that the Pd and Pt cores wrapped by 1ML wide Ge and Si shells show linear I–V characteristics behaviour as shown in Fig. 4a. The value of current passing through the SR increases linearly with applied voltage. The cores wrapped with Si-shell show higher current value than Ge-shell at corresponding applied voltage. The current is calculated to 140 μA and 127 μA for Pd/Si (1R) and Pt/Si (1R), respectively at 1.0 V, while the Pd/Ge and Pt/Ge shows the current value of 75.3 μA and 68.5 μA, respectively. It is also observed that these NWs show a high magnitude of current at corresponding applied voltage comparing to Sb^29^, As^29^ and Ni^30^ core, wrapped with same shell material and same diameter.

Further, when the core is wrapped 2ML wide shell, the magnitude of current of the core–shell NWs abruptly increases, as shown in Fig. 4b. Both Pd/Si (2R) and Pt/Si (2R) NWs show an increment of current of around 180% and reach 355 μA and 392 μA, respectively for applied voltage 1.0 V. The current value for Pd/Ge (2R) and Pt/Ge (2R) NW increases upto 226 μA and 113 μA at applied voltage 1.0 V, respectively as the shell diameter increases. The increasing current value corresponding to applied voltage with increasing the diameter of the NW leads to decrease in resistivity of the NW. This phenomenon can be explained by Fuchs–Sondheimer (FS) theory, as the size of our core–shell NWs is shorter than the mean free path of the electron, therefore surface scattering will influence the resistance of the NW, which leads to decrease in resistivity^27,28^.

### Optical properties

**Figure 5:**
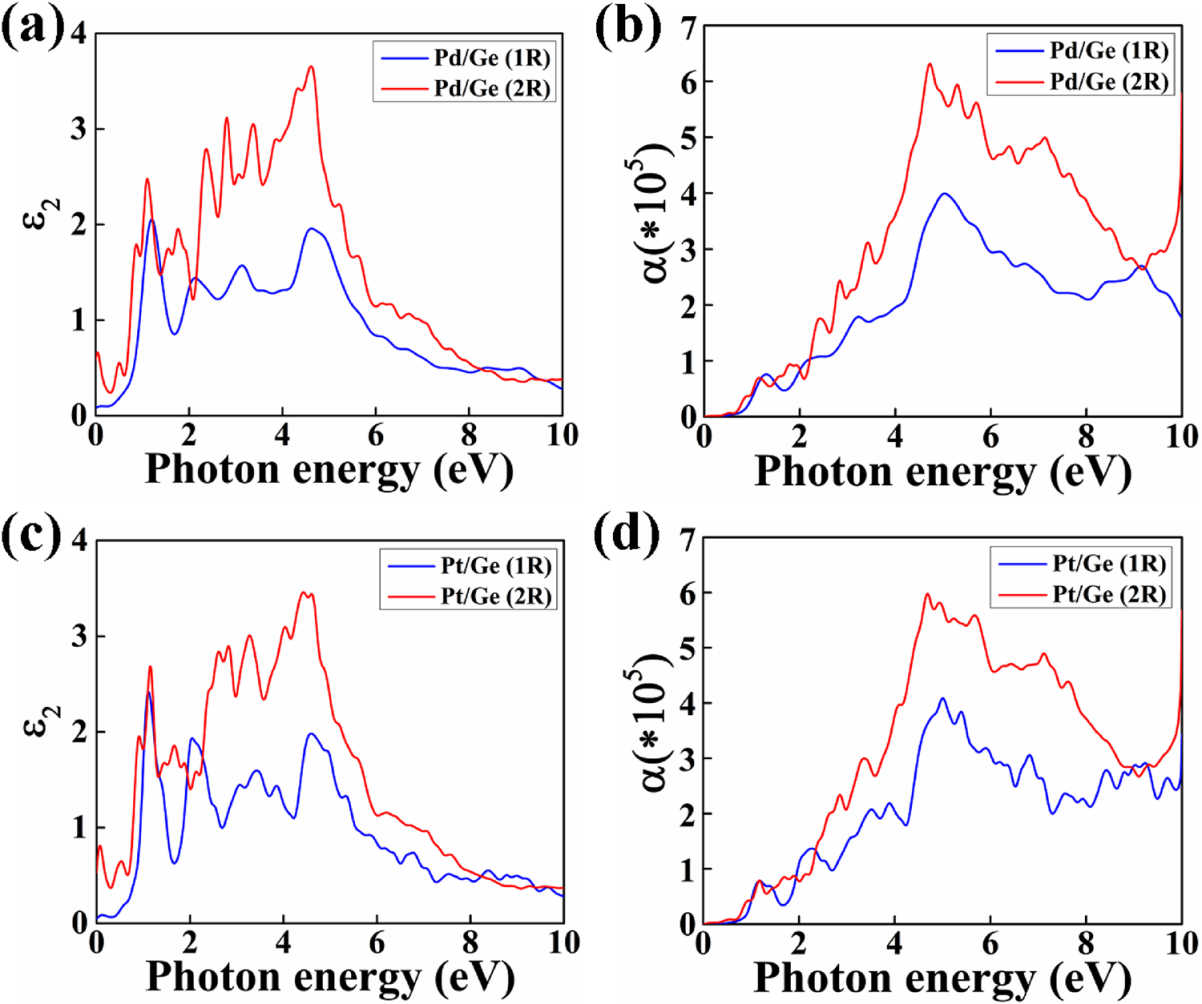
The diameter dependent imaginary part of the dielectric constant (Ɛ_2_) with photon energy and its corresponding adsorption co-efficient are shown in (**a**,**b**) for Pd/Ge and (**c**,**d**) for Pt/Ge core/shell NW.

**Figure 6:**
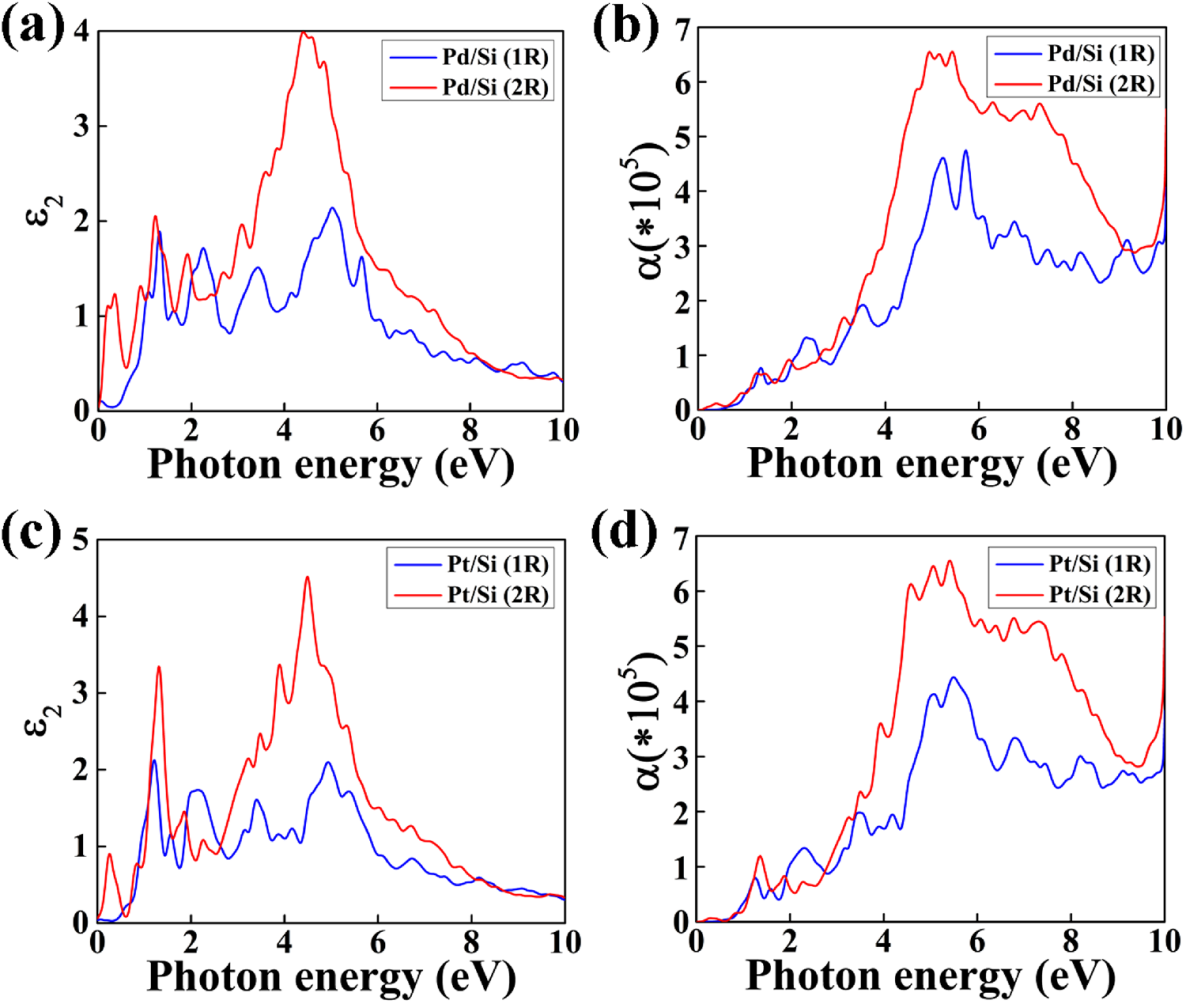
The diameter dependent imaginary part of the dielectric constant (Ɛ_2_) with photon energy and its corresponding adsorption co-efficient are shown in (**a**,**b**) for Pd/Si and (**c**,**d**) for Pt/Si core/shell NW.

The imaginary part (Ɛ_2_) of the frequency dependent complex dielectric function can be described by the electronic band structure of the material and is related to the absorption spectra. We have investigated the optical properties of the nanowires in plane (E||Z) polarization of light. The observed peaks in the Ɛ_2_ graph provide the information about the interband transitions of electron corresponding to the photon energy from occupied states to unoccupied states of the band structure. The Ɛ_2_ of the NWs are shown in Figs. 5a,c and 6a,c. We have observed a valley region upto 1 eV for all the NWs having diameter of 1R.

However, in case of NWs with 2R diameter, sharp peaks have been observed in that valley region. These peaks are obtained due to the metallic behaviour of the NWs with 2R diameter. We can clearly observe the transition from semi-metallic to metallic behaviour in Ɛ_2_ graph due to the transition of electrons.

Further, we have studied absorption coefficient of the NWs to analyze their potential in optical device applications. It provides the information of the rate at which intensity of light decreases as it passes through the material. These core/shell NWs show high absorption co-efficient in the ultraviolet (UV) range and the absorption co-efficient increases with increase in diameter of the NWs as shown in Figs. 5b,d and 6b,d. This absorption co-efficient of the material depends on the imaginary part (Ɛ_2_) of the frequency dependent complex dielectric function. High absorption co-efficient of 3.9 × 10^5^ cm^−1^ is obtained at 5.04 eV for Pd/Ge NW with 1R diameter. However, as the diameter of the NW increase to 2R, the absorption co-efficient red shifted to 4.69 eV and the value increases to 6.25 × 10^5^ cm^−1^. Similarly, Pt/Ge (1R) shows highest peak at 4.99 eV of 4.0 × 10^5^ cm^−1^ and increases to 5.98 × 10^5^ cm^−1^ at 4.68 eV for 2R diameter. The Pd/Si (1R) and Pt/Si (1R) NWs exhibit absorption co-efficient of 4.68 × 10^5^ cm^−1^ at 5.71 eV and 4.41 × 10^5^ cm^−1^ at 5.49 eV, respectively. The value of co-efficient changes to 6.53 × 10^5^ cm^−1^ at 5.42 eV for Pd/Si (2R) and 6.54 × 10^5^ cm^−1^ at 5.41 eV for Pt/Si (2R) NW. The peaks move to lower energies as diameter increases is due to quantum confinement effect^31^.

### Nanowire as optical sensor

Gas detection has become as field of continuous research to detect industrial and automobile emissions and for monitoring our environment and household safety. In this regard, metallic NWs like, Au^32^ and Pd^17^ have shown great possibility in the field of gas sensing. This motivates us to investigate the optical response of Pd and Pt based core–shell NWs in O_2_ and CO_2_ environment.

### Adsorption of gas molecules

**Figure 7:**
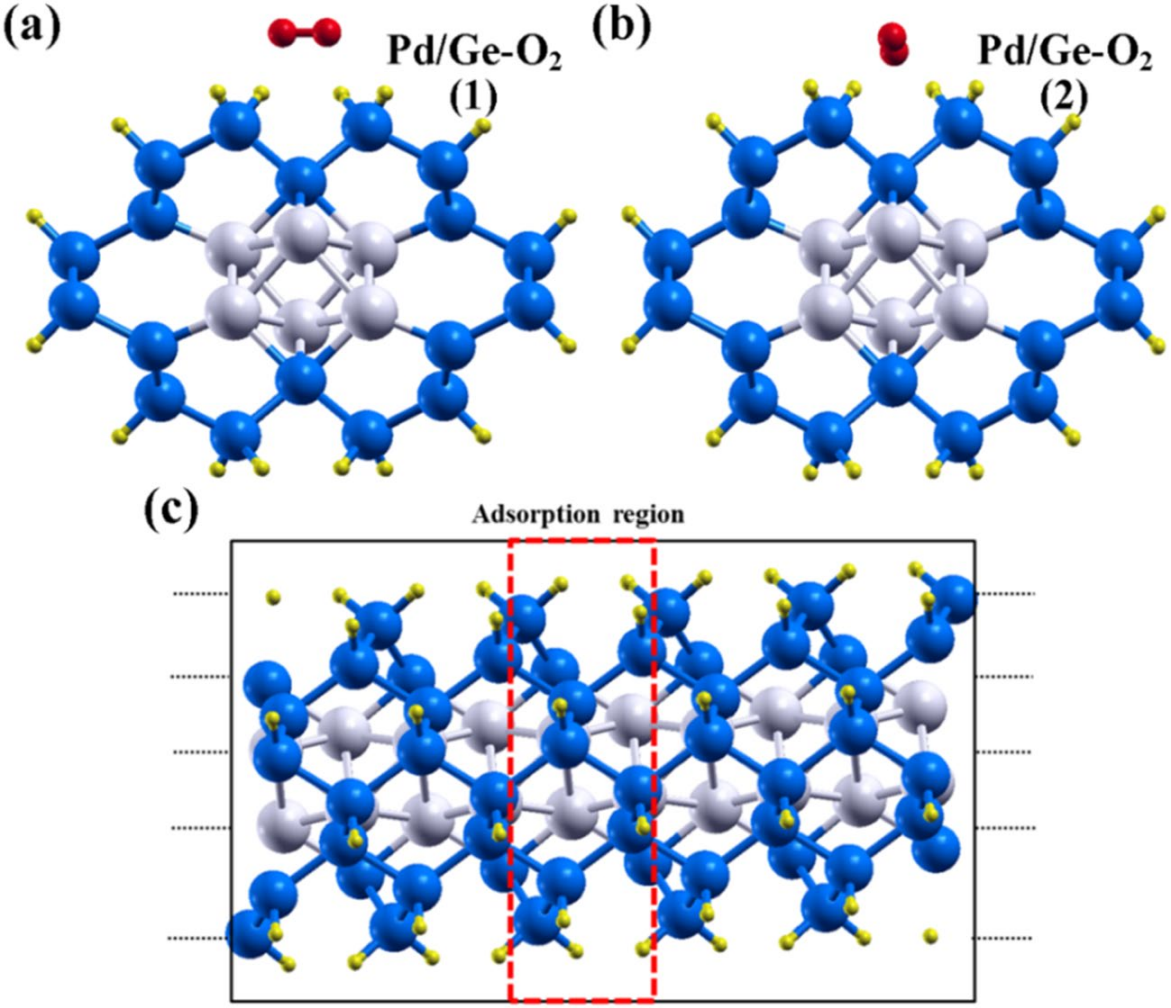
Relaxed O_2_ gas molecules on Pd/Ge-NW having configuration (**a**) type 1 geometry and (**b**) type 2 geometry. Schematic view of NW system having 5 primitive cells along the z-axis is shown in figure (**c**).

In this section, we have focused our study on the effect of adsorption gas molecules on the optical properties of the core–shell nanowires. To reveal the possibilities of Pd and Pt core–shell NW based sensors, it is important to understand the interaction between the NW and adsorbent gas molecules. We have considered two types of configuration gas molecule geometry, represented by 1 and 2 as shown in Fig. 7a,b. We have adsorbed O_2_ and CO_2_ gas molecules on the (1 × 1 × 3) supercell of 1R core–shell NW as shown in Fig. 7c. The gas molecules are placed on the surface of the NW at a distance of 1.5 Å. After relaxation, gas molecules are observed to be changed their positions as shown in Figures S4, S5. The distance between the gas molecule from the surface of the NW after relaxation are detailed in Table 2. The change in their positions and structure of gas molecules is observed due to the interaction between two. According to these changes, we have calculated adsorption energy and relaxation time of the molecules.

**Table 2:** Calculated distance between the nanowire and gas molecules, adsorption energy and relaxation time of the gas molecules.

Nanowire	Adsorbed gas molecules	Position of the molecules	Distance between the nanowire and gas molecules (Å)	Adsorption energy (eV)	Relaxation time
Pd/Ge	CO_2_	1	2.79	− 0.18	1.0 × 10^3^
		2	2.62	− 0.20	2.2 × 10^3^
	O_2_	1	2.32	− 0.45	3.5 × 10^7^
		2	2.02	− 0.56	2.4 × 10^9^
Pt/Ge	CO_2_	1	2.58	− 0.19	1.5 × 10^3^
		2	2.70	− 0.21	3.3 × 10^3^
	O_2_	1	2.08	− 0.50	2.4 × 10^8^
		2	1.99	− 0.51	3.6 × 10^8^
Pd/Si	CO_2_	1	2.66	− 0.20	2.2 × 10^3^
		2	2.60	− 0.20	2.2 × 10^3^
	O_2_	1	2.10	− 0.65	7.9 × 10^10^
		2	1.98	− 0.62	2.5 × 10^10^
Pt/Si	CO_2_	1	2.70	− 0.20	2.2 × 10^3^
		2	2.61	− 0.22	4.9 × 10^3^
	O_2_	1	2.30	− 0.43	1.6 × 10^7^
		2	1.99	− 0.57	3.6 × 10^9^

The adsorption energy of the gas molecules on the NW is calculated from the equation;

6 $$E_{ads} = E_{adsorbed - NW} - (E_{core/shell} + E_{adsorbent - gas} )$$

where, $E_{adsorbed - NW}$ is the total energy of the gas molecule adsorbed on the core–shell NW, $E_{core/shell}$ is the total energy of the respective core–shell NW and $E_{adsorbent - gas}$ is the total energy of the respective gas molecule. We have observed that CO_2_ gas molecules on core–shell NW show adsorption energy of − 0.18 ~ − 0.22 eV. However, O_2_ gas molecule shows comparatively higher adsorption energy of − 0.45 ~ − 0.65 eV. It is noticed that the type 1 geometry of O_2_ gas molecule on Pd/Si-NW shows highest adsorption energy of − 0.65 eV as shown in Table 2. Another important feature to study is the relaxation time of the molecules, which gives information about the sustaining time period of gas molecules on the NWs. The relaxation time is calculated by;

7 $$\tau \approx \exp ({{ - E_{ads} } \mathord{\left/ {\vphantom {{ - E_{ads} } {K_{B} T)}}} \right. \kern-\nulldelimiterspace} {K_{B} T)}}$$

where, $E_{ads}$ is the calculated adsorption energy, $K_{B}$ is the Boltzmann constant and T is the temperature. We have considered temperature to be 300 K in this calculation. As the relaxation time is directly proportional to the adsorption energy, we have observed that relaxation time for O_2_ gas molecule on the NW is greater the CO_2_ gas. Similarly, the type 1 geometry of O_2_ gas on Pd/Si NW shows maximum relaxation time of 7.9 × 10^10^.

### Electronic properties

**Figure 8:**
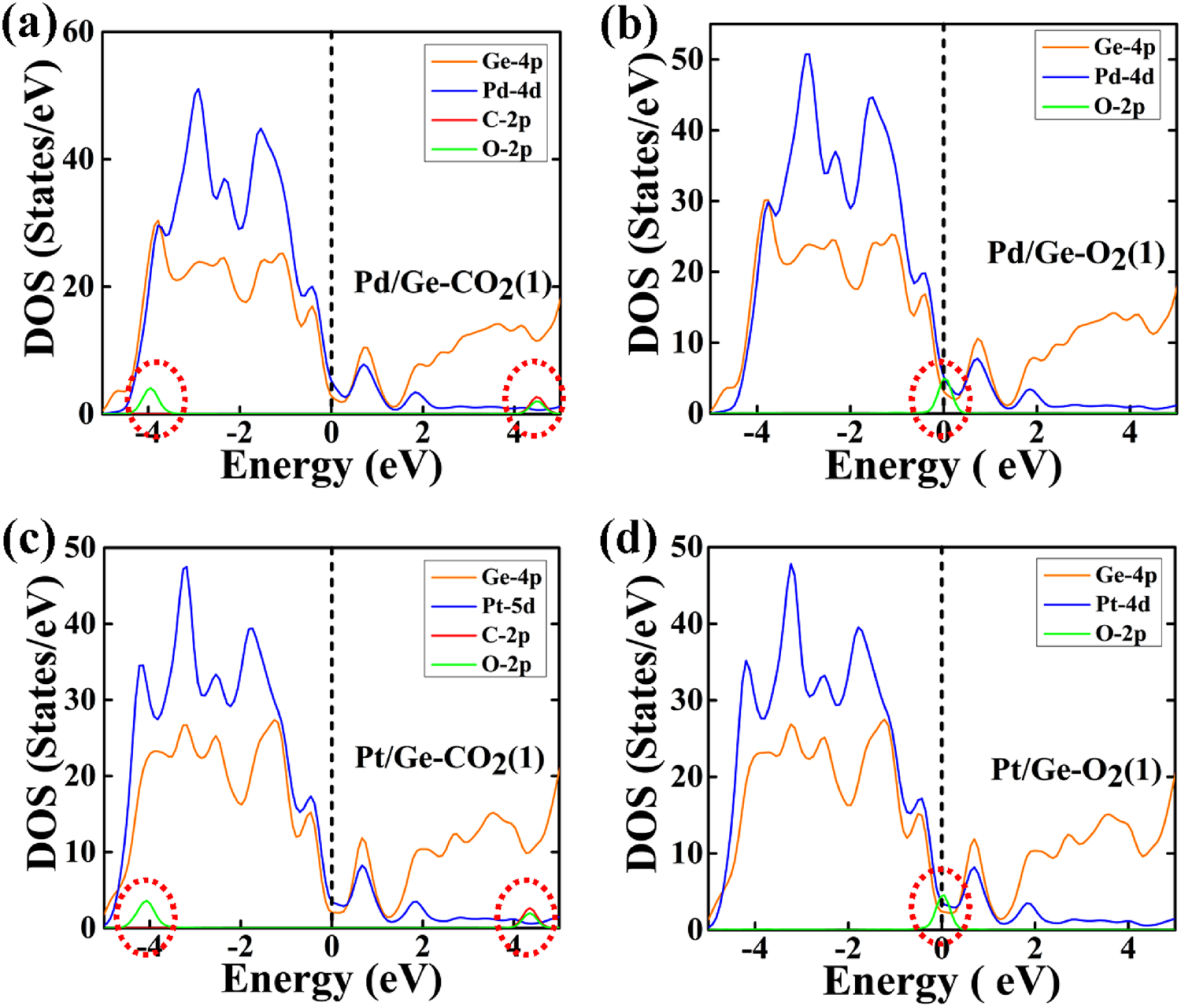
The partial density of states (PDOS) of type 1 geometry of CO_2_ and O_2_, are adsorbed on Pd/Ge and Pt/Ge core–shell NW: (**a**) CO_2_ adsorbed Pd/Ge NW, (**b**) O_2_ adsorbed Pd/Ge NW, (**c**) CO_2_ adsorbed Pt/Ge NW and (**d**) O_2_ adsorbed Pt/Ge NW. The Fermi energy is set at 0 eV.

**Figure 9:**
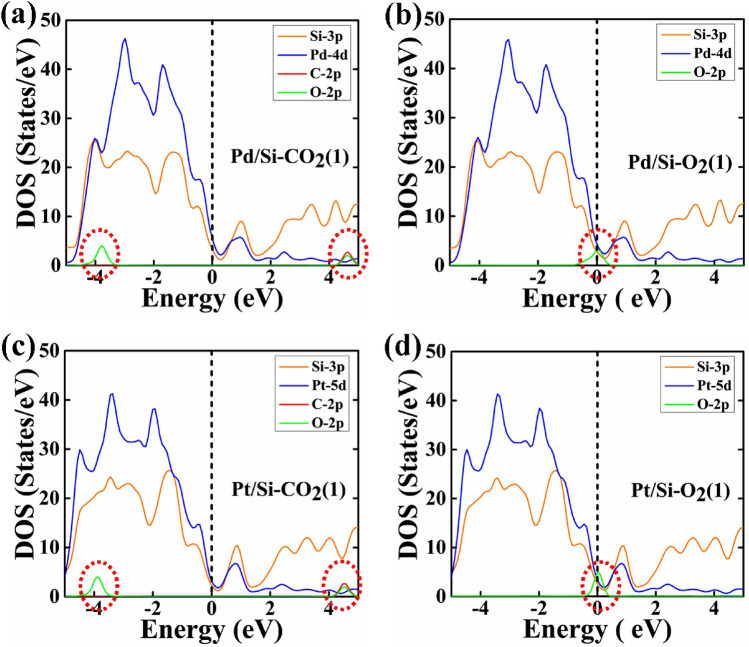
The partial density of states (PDOS) of type 1 geometry of CO_2_ and O_2_, are adsorbed on Pd/Si and Pt/Si core–shell NW: (**a**) CO_2_ adsorbed Pd/Si NW, (**b**) O_2_ adsorbed Pd/Si NW, (**c**) CO_2_ adsorbed Pt/Si NW and (**d**) O_2_ adsorbed Pt/Si NW. The Fermi energy is set at 0 eV.

The study of electronic properties of the gas adsorbed nanowire is very important to understand the interaction between them; therefore, we have computed projected density of states (PDOS) to analyze the contribution gas molecules near the Fermi energy level and are shown in Figs. 8, 9 and Figure S6 and S7.

The contribution of O_2_ gas molecules is observed at the Fermi energy level. Therefore, O_2_ gas molecule shows stronger adsorption energy and higher relaxation time than CO_2_ molecule. Another smaller peak of O_2_ is also observed near the Fermi levels in the range of 0.65–0.75 eV, which is not visible in Figs. 8 and 9. In the case of CO_2_ adsorbed core–shell nanowires, the PDOS reveals the contribution of O-2p orbitals of CO_2_ in the valance band from ~ − 3.5 eV to ~ − 4.5 eV. However, at conduction band, the contribution of CO_2_ is observed due to the hybridization of C-2p and O-2p orbital in the range from ~ 4.0 eV to ~ 4.7 eV. The range and states/eV of O_2_ and CO_2_ contribution slightly changes with different core–shell NW and geometry of the gas molecules.

### Effect on optical properties

We have clearly observed that CO_2_ gas molecules adsorbed on NWs shows absorption energy in the range of − 0.18 ~ − 0.22 eV. It is reported that the interaction between the gas molecules and the substrate should be in the scale of − 0.5 eV for effective sensing^33^. Therefore, we will focus on O_2_ gas molecules adsorbed on the core/shell NWs as the absorption energies are in the range of − 0.45 ~ − 0.65 eV.

**Figure 10:**
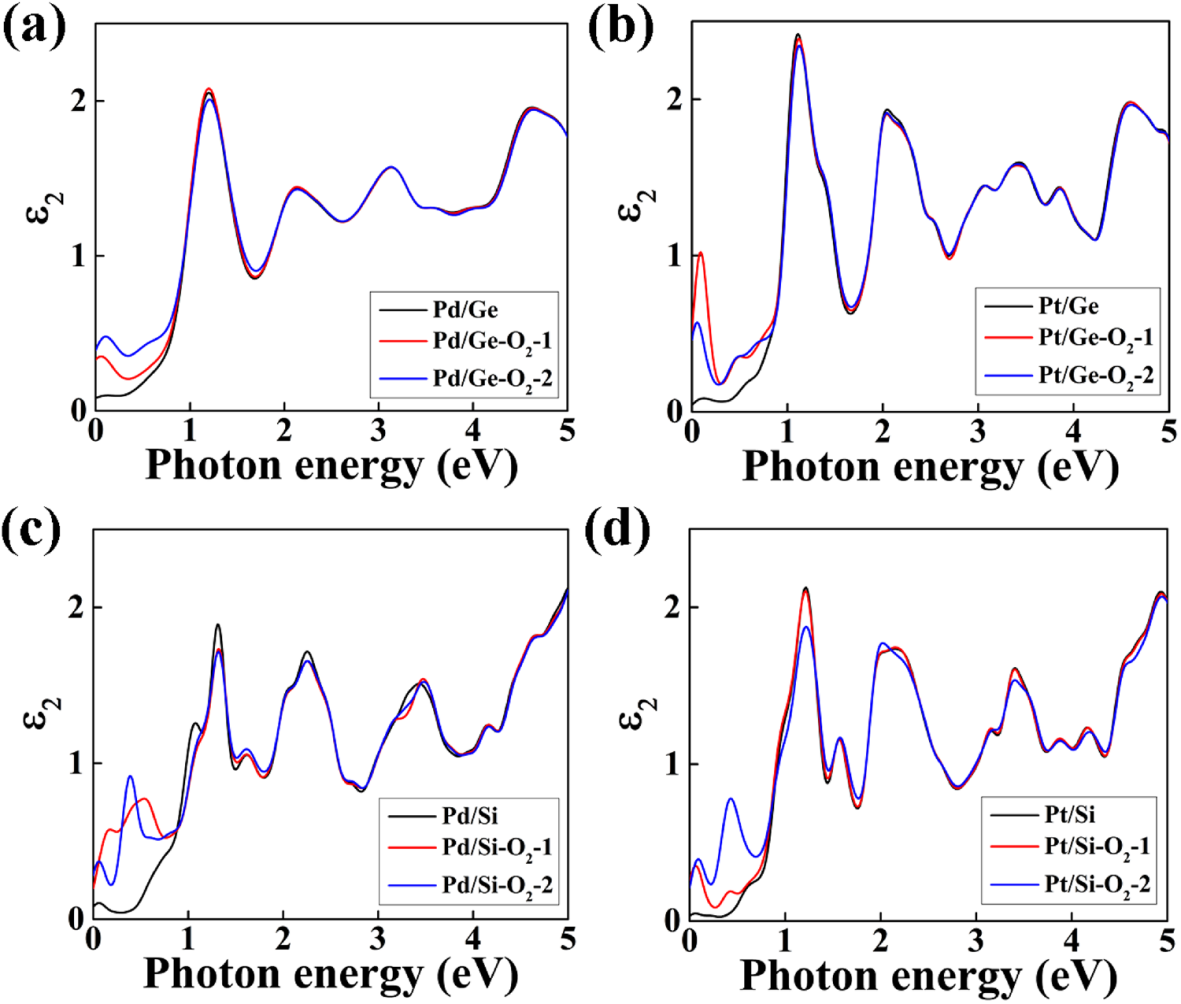
Variation of imaginary part (Ɛ_2_) of the dielectric function between the O_2_ adsorbed (**a**) Pd/Ge, (**b**) Pt/Ge, (**c**) Pd/Si and (**d**) Pt/Si NW and their corresponding pure NW.

We have analyzed imaginary part (Ɛ_2_) of the dielectric function of O_2_ gas adsorbed NWs and compared the results with pure one as shown in Fig. 10. We have calculated the imaginary part for the both type 1 and 2 geometry configuration of O_2_ gas molecules. From Fig. 10a, we have observed a valley region upto 1 eV, there are no sharp peaks. However, in presence of O_2_ gas molecules, the Pd/Ge core/shell NW shows a peak at 0.007 eV for type 1 geometry configuration and at 0.11 eV for type 2 geometry configuration. The contribution of O_2_ gas molecule is observed in infra-red (IR) region. We have observed no change in optical properties in the visible and ultraviolet (UV) range. The extra peak observed in the IR region is due to O-2p states electronic contribution at the Fermi energy level as shown in Fig. 8b and ESI, Figure S6 (b). Similarly, we have observed imaginary part peak for O_2_ adsorbed Pt/Ge NW at IR-region due O-2p states electron contribution as comparison to flat valley for pure NW (Fig. 10b). In case of Pd/Si NW, two peaks are observed at 0.16 eV and 0.55 eV for type 1 position and at 0.04 eV and 0.37 eV for type 2 configuration of O_2_ gas (Fig. 10c). There is no change in optical properties of Pd/Si-NW in the visible and ultraviolet (UV) range in presence of O_2_ gas molecule. We have also observed two peaks for O_2_ adsorbed Pt/Si-NW. The contribution of O_2_ gas is observed at 0.08 eV and 0.40 eV for type 1 and at 0.09 eV and 0.43 eV for type 2 configuration as shown in Fig. 10d. It can be clearly noted that the change in optical properties in core–shell NWs is observed at IR region due to the O-2p orbital electron contribution at the Fermi energy level. The optical properties of the core/shell NWs indicate that these have great potential applications in optical gas sensors for sensing O_2_ gas in the IR region.

## Conclusions

In this work, we have investigated a new metallic core–shell nanowire geometry of that could be obtained experimentally, that is silicon (Si) and germanium (Ge) nanowires with cores constituted by group-10 element Pd and Pt. We have fully optimized four different core–shell NW configurations and negative values of cohesive energy ($E_{coh}$) indicates the stability of the nanowires. The electronic band structure properties show transition from semi-metallic to metallic behaviour with the diameter of the nanowire. The linear current–voltage (IV) characteristics of the NWs indicate ohmic behaviour and it has been found that current values in accordance with applied voltage show strong dependence on the diameter of the NWs. We have also studied diameter dependent optical properties. The absorption co-efficient peaks move to lower energies is due to quantum confinement effect. Furthermore, the change in optical properties of the NWs in presence of O_2_ and CO_2_ environment shows that Pd and Pt based core/shell NWs have potential as a gas sensor. These core–shell nanowires with tunable transport properties show a potential for applications as electron connectors in nanoelectronic devices as well as nano gas detector for detecting O_2_ gas.

## Supplementary Information

Supplementary Information

## Data Availability

The authors declare that all data supporting the findings of this study are available within the paper and its supplementary information files.
