# Supplementary material for: Metallic one-dimensional heterostructure for gas molecule sensing
Source: Sci Rep. 2021 Jan 11;11:433. doi: 10.1038/s41598-020-79921-8 (PMC7801624; doi:10.1038/s41598-020-79921-8)
Supplement: Supplementary file 1 — Supplementary Information [file 41598_2020_79921_MOESM1_ESM.docx]

**Electronic supplementary information (ESI)**

**Metallic one-dimensional heterostructure for gas molecule sensing**

Prabal Dev Bhuyan^1, 2^_,_ Sanjeev K. Gupta^1,*^, Rajeev Ahuja^3, 4^ and P. N. Gajjar^2,*^

^1^Computational Materials and Nanoscience Group, Department of Physics and Electronics,

St. Xavier's College, Ahmedabad 380009, India

^2^Department of Physics, Gujarat University, Ahmedabad 380009, India

^3^Condensed Matter Theory Group, Department of Physics and Astronomy,

Box 516, Uppsala University, S-75120 Uppsala, Sweden

^4^Applied Materials Physics, Department of Materials and Engineering, Royal Institute of Technology (KTH), S-100 44 Stockholm, Sweden

*(November 1, 2020)*

**Keyword(s):** Nanowires, Density functional theory, Electronic properties, Optical properties, Gas sensing

***Corresponding author**: Dr Sanjeev K. Gupta (E-mail: sanjeev.gupta@sxca.edu.in) and Prof. P. N. Gajjar (pngajjar@gujaratuniversity.ac.in)


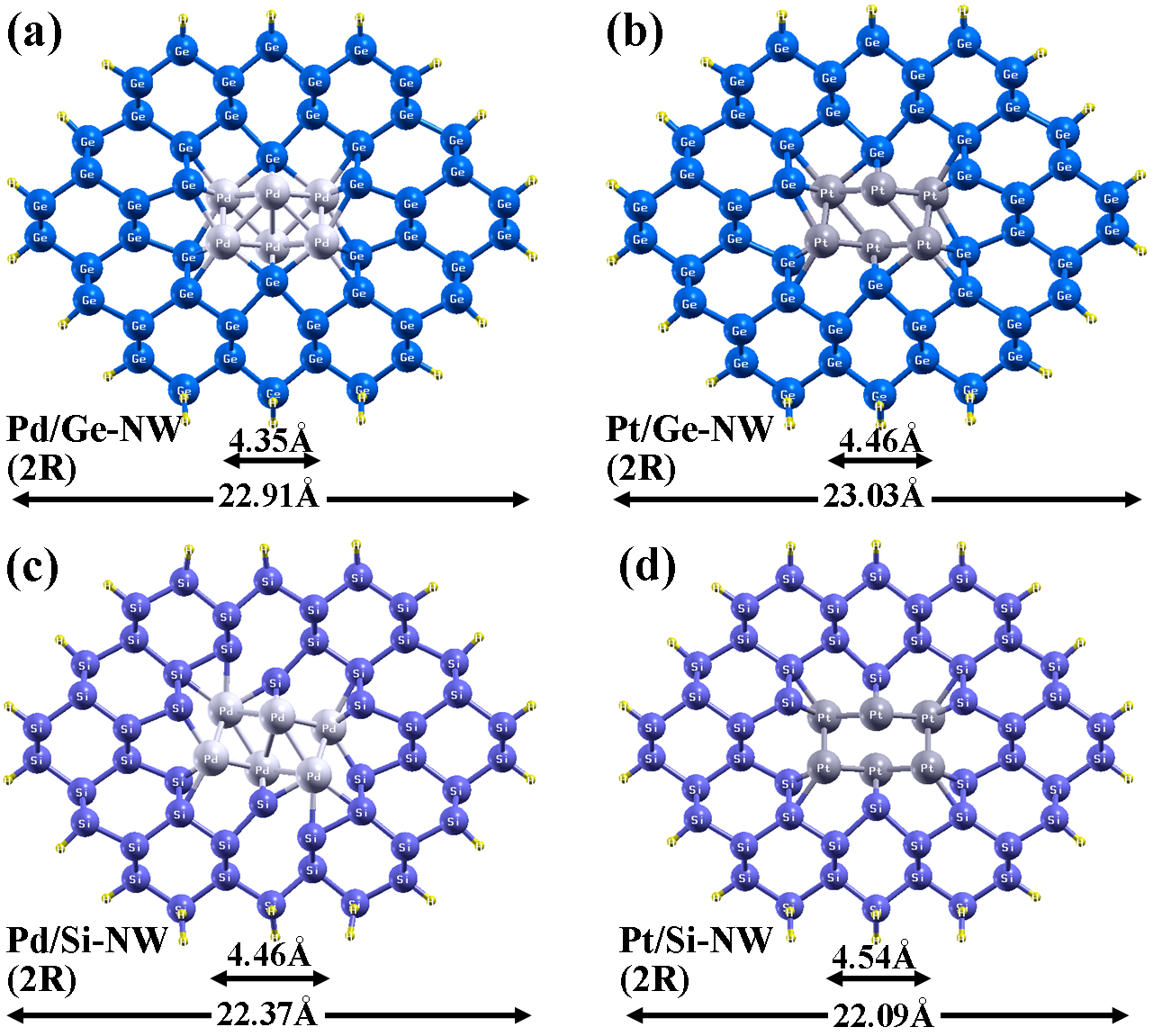


**Figure S1:** Front view of the fully optimized structures of (a) Pd/Ge, (b) Pt/Ge, (c) Pd/Si and (d) Pt/Si core-shell NWs. The core is wrapped by 1ML Ge or Si-shell. Hydrogen atoms are passivated on the surface of the NWs to prevent the daggling bonds.


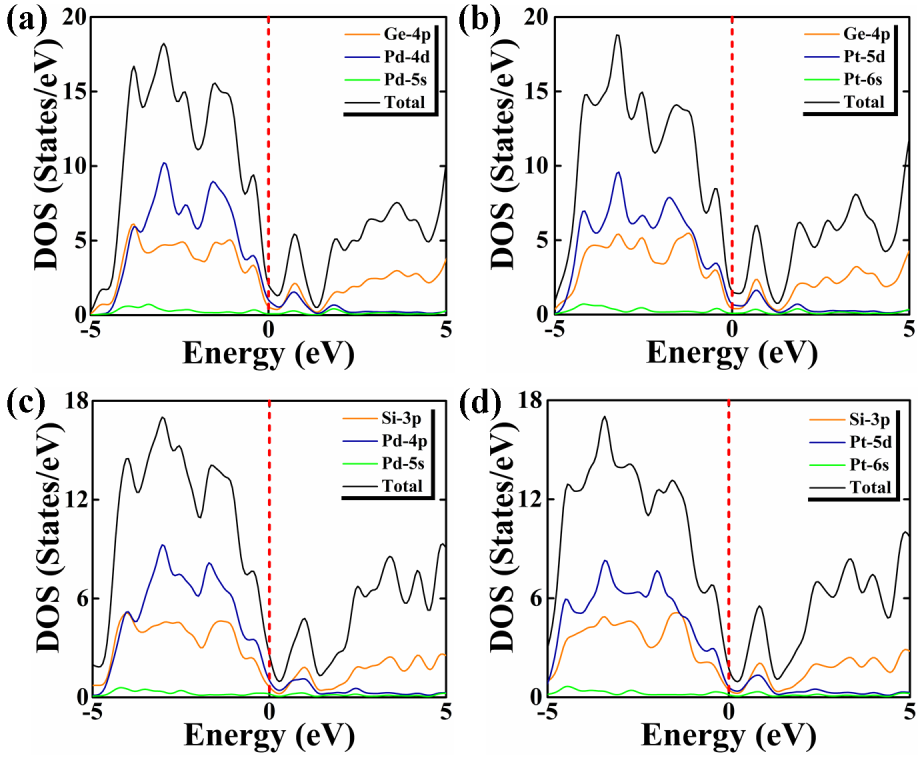


**Figure S2:** The partial density of states (PDOS) of 1R core-shell NWs (a) Pd/Ge, (b) Pt/Ge, (c) Pd/Si and (d) Pt/Si.


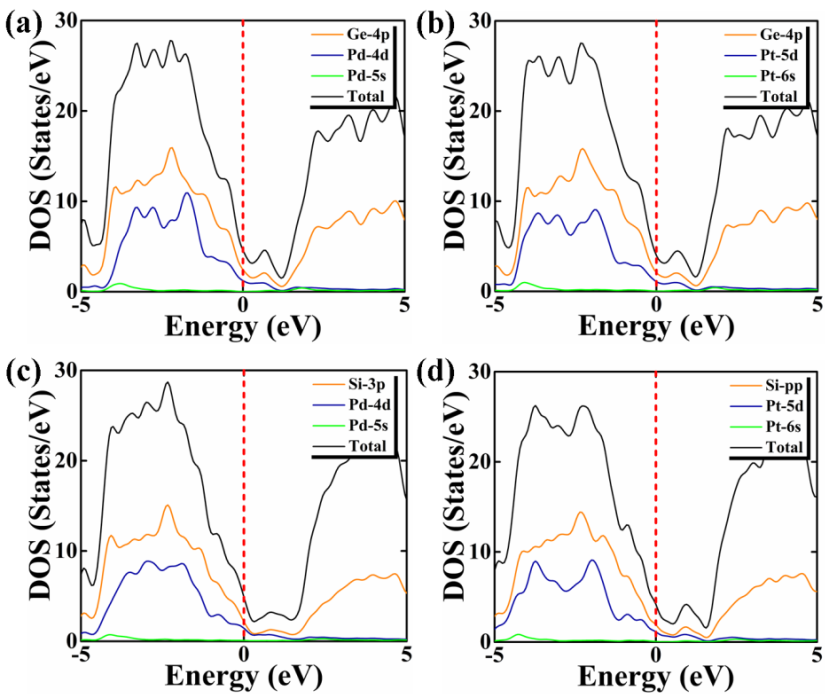


**Figure S3:** The partial density of states (PDOS) of 2R core-shell NWs (a) Pd/Ge, (b) Pt/Ge, (c) Pd/Si and (d) Pt/Si.


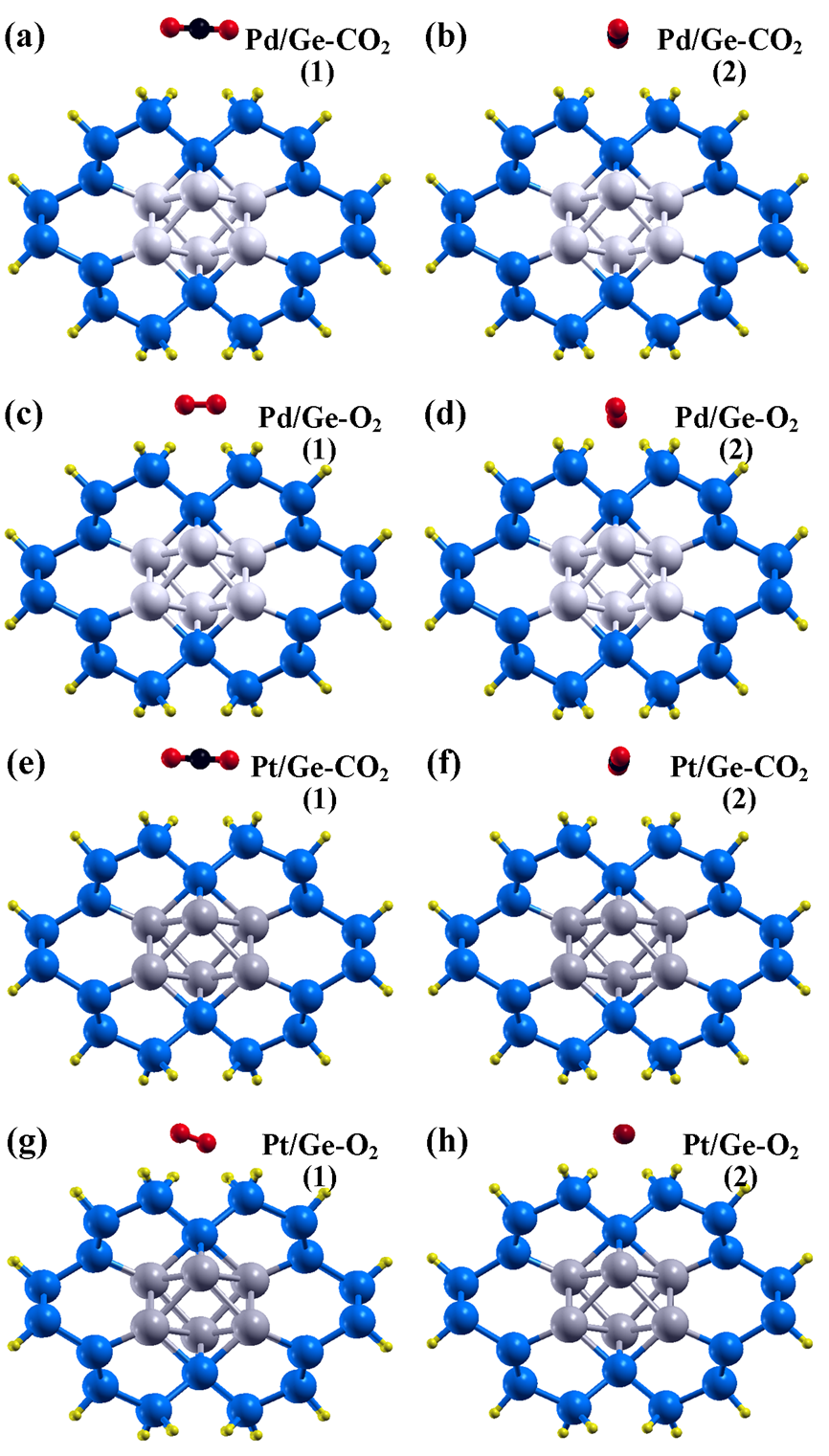


**Figure S4:** Side view of the relaxed gas molecules O_2_ and CO_2_ on Pd and Pt core, wrapped by Ge shell, NWs. (a, b) shows CO_2_ adsorbed Pd/Ge NW, (c, d) shows O_2_ adsorbed Pd/Ge NW, (e, f) shows CO_2_ adsorbed Pt/Ge NW and (g, h) shows O_2_ adsorbed Pt/Ge NW. We have considered two types of configuration gas molecule geometry, represented by 1 and 2.


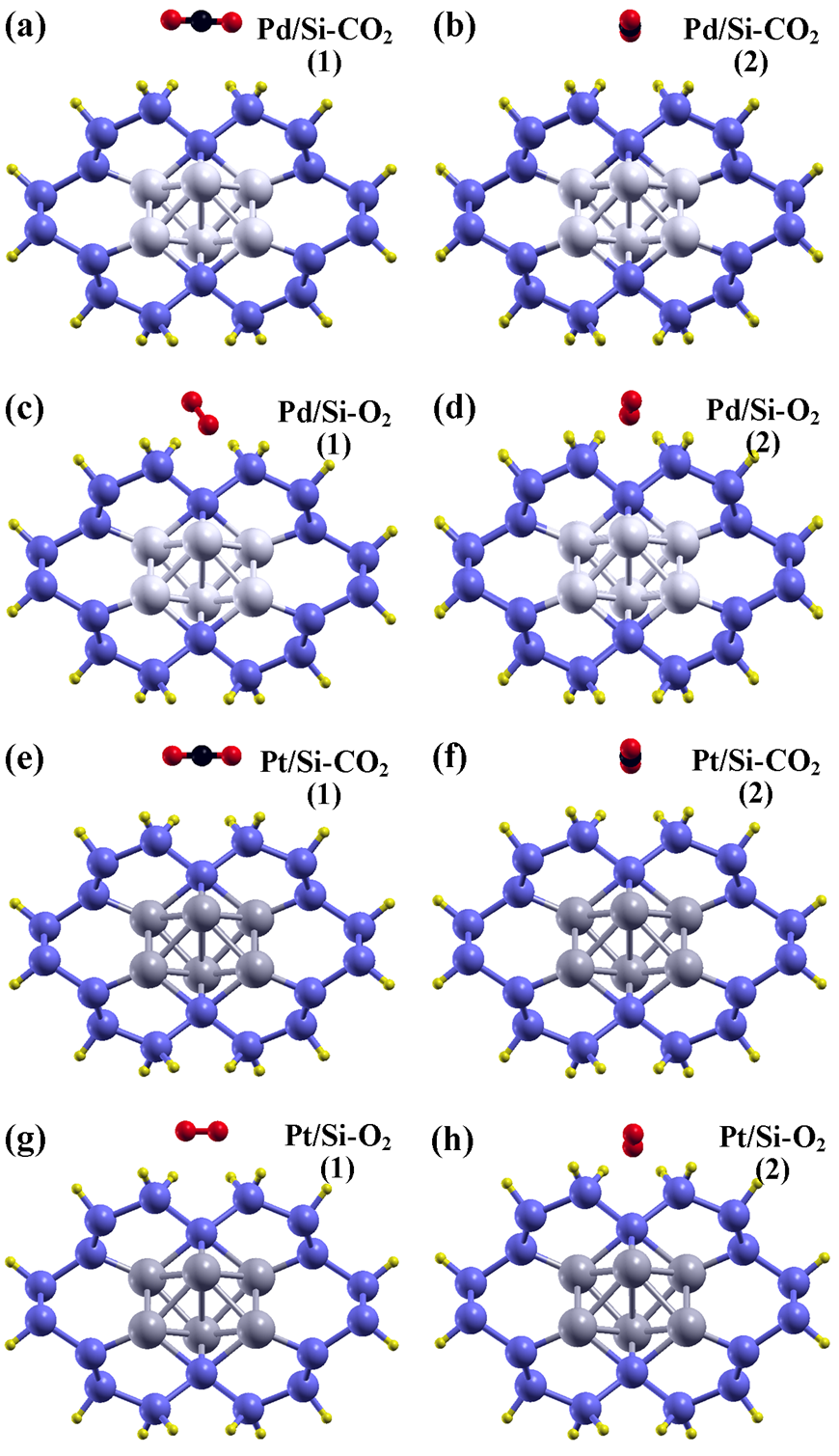


**Figure S5:** Side view of the relaxed gas molecules O_2_ and CO_2_ on Pd and Pt core, wrapped by Si shell, NWs. (a, b) shows CO_2_ adsorbed Pd/Si NW, (c, d) shows O_2_ adsorbed Pd/Si NW, (e, f) shows CO_2_ adsorbed Pt/Si NW and (g, h) shows O_2_ adsorbed Pt/Si NW. We have considered two types of configuration gas molecule geometry, represented by 1 and 2.


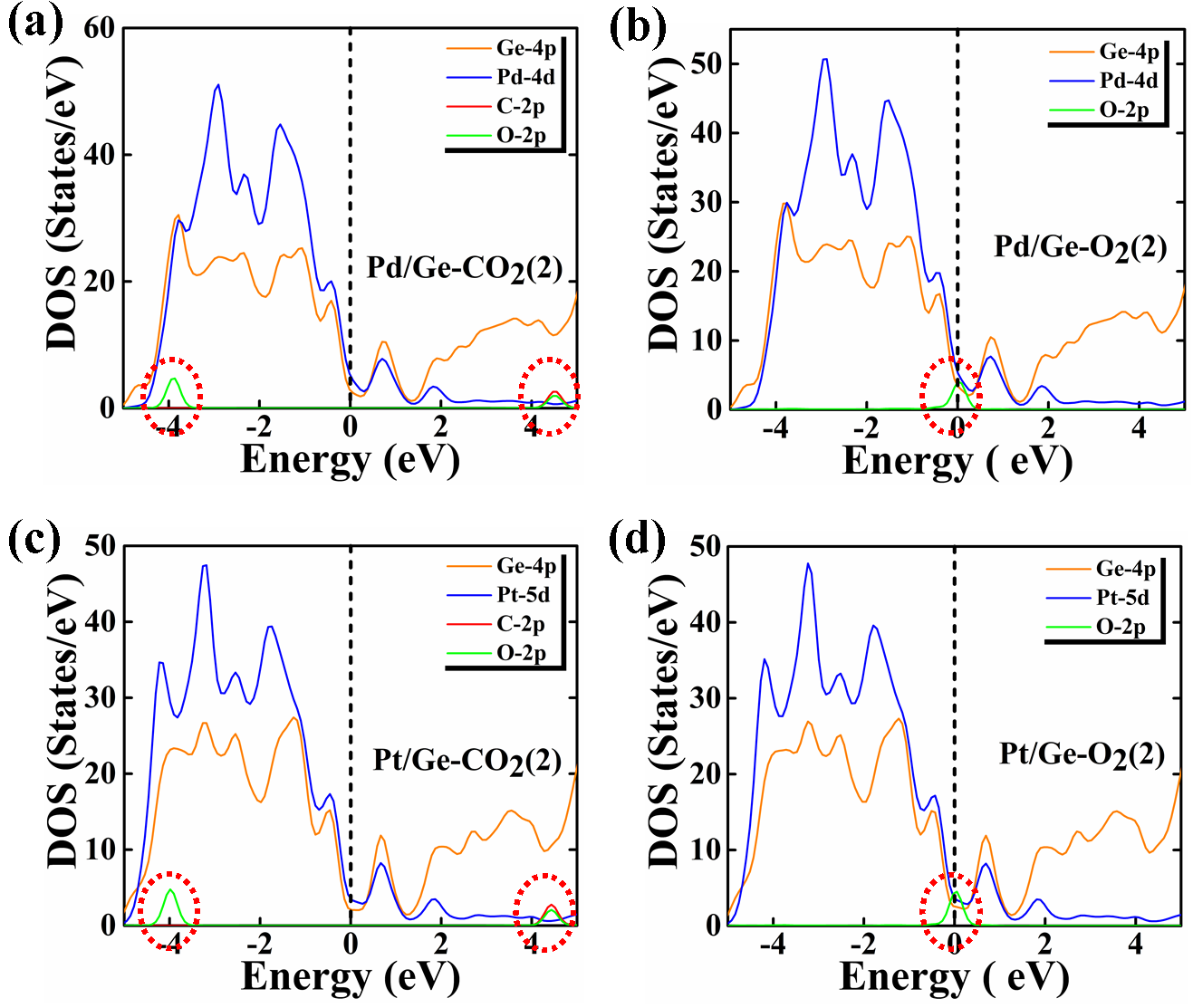


**Figure S6:** The partial density of states (PDOS) of type 2 geometry of CO_2_ and O_2_, are adsorbed on Pd/Ge and Pt/Ge core-shell NW: (a) CO_2_ adsorbed Pd/Ge NW, (b) O_2_ adsorbed Pd/Ge NW, (c) CO_2_ adsorbed Pt/Ge NW and (d) O_2_ adsorbed Pt/Ge NW. The Fermi energy is set at 0eV.


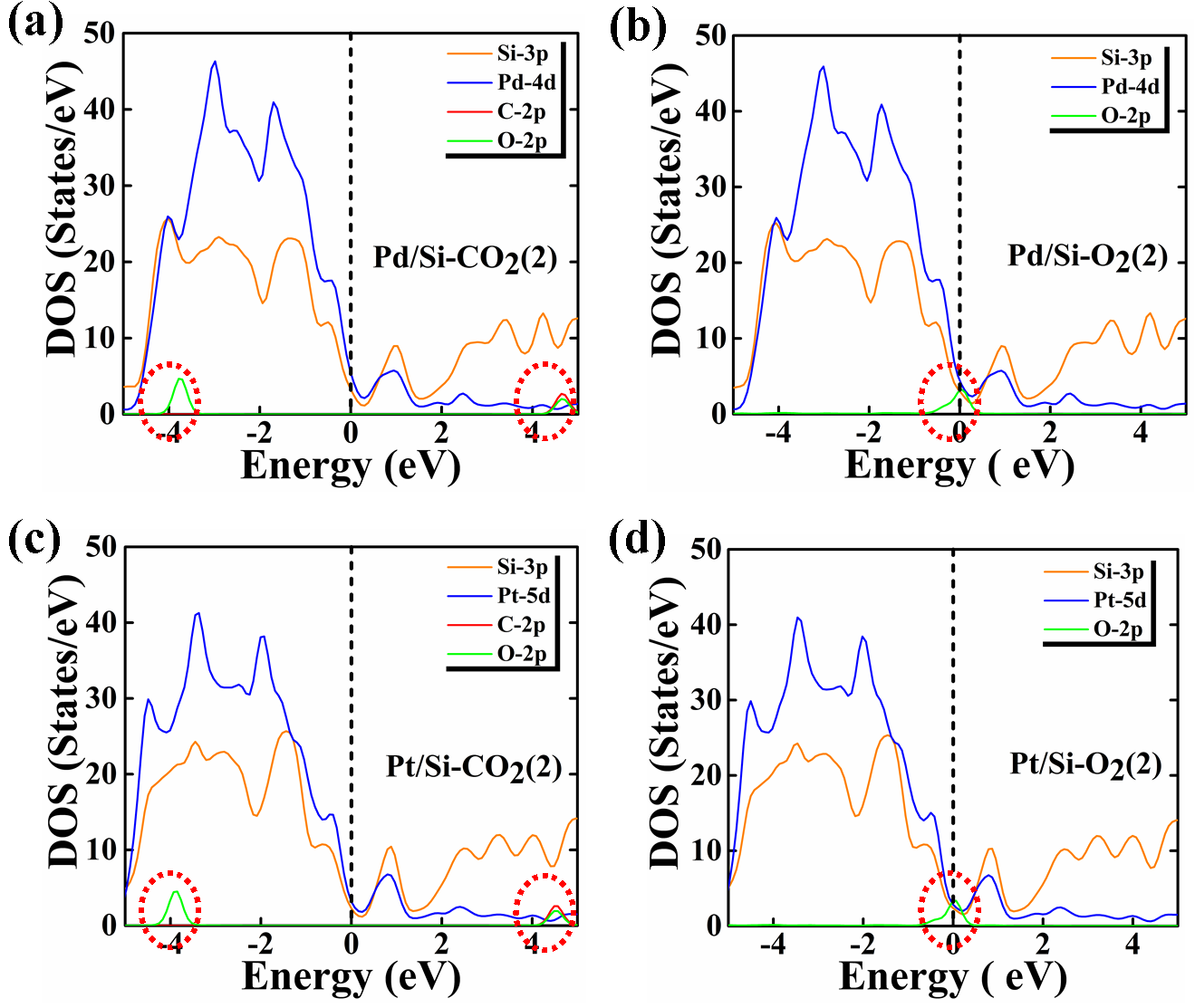


**Figure S7:** The partial density of states (PDOS) of type 2 geometry of CO_2_ and O_2_, are adsorbed on Pd/Si and Pt/Si core-shell NW: (a) CO_2_ adsorbed Pd/Si NW, (b) O_2_ adsorbed Pd/Si NW, (c) CO_2_ adsorbed Pt/Si NW and (d) O_2_ adsorbed Pt/Si NW. The Fermi energy is set at 0eV.

**
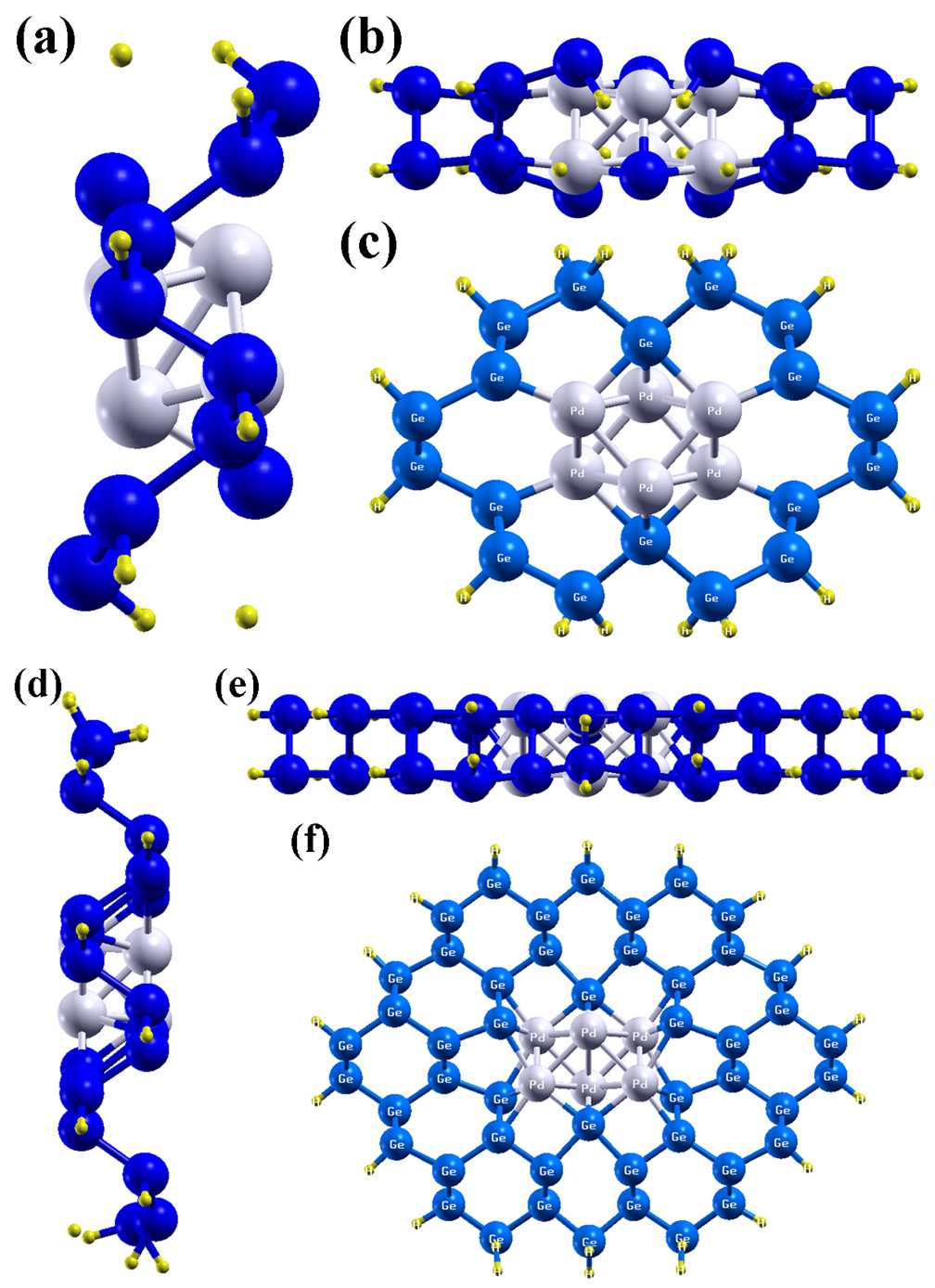
**

**Figure S8:** (a) Side view, (b) top view and (c) front view of the fully optimized structures of Pd/Ge-NW of diameter 1.4~1.5Å. Again, (d) side view, (e) top view and (f) front view of the fully optimized structures of Pd/Ge-NW of diameter 2.2~2.3Å.


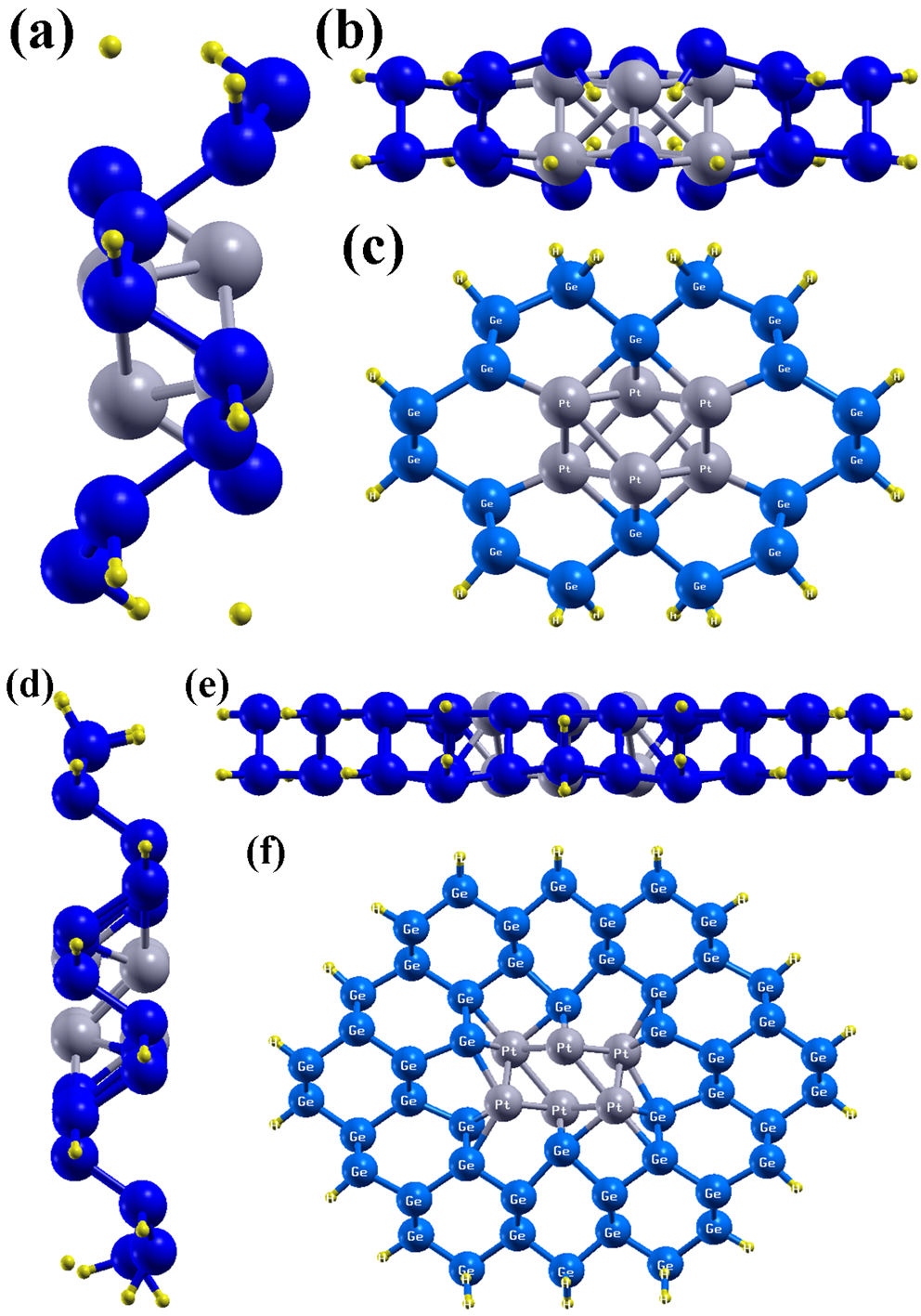


**Figure S9:** (a) Side view, (b) top view and (c) front view of the fully optimized structures of Pt/Ge-NW of diameter 1.4~1.5Å. Again, (d) side view, (e) top view and (f) front view of the fully optimized structures of Pt/Ge-NW of diameter 2.2~2.3Å.


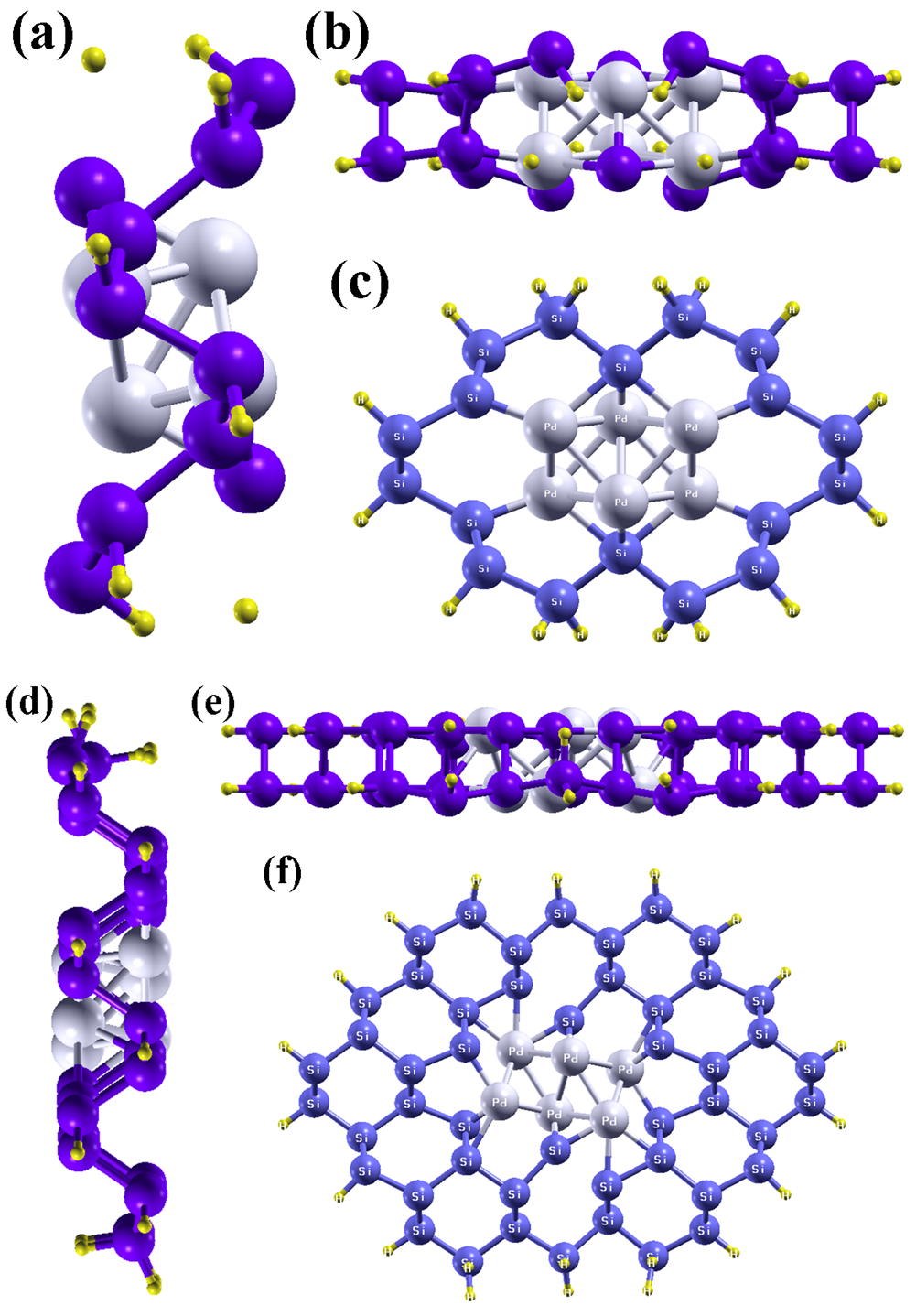


**Figure S10:** (a) Side view, (b) top view and (c) front view of the fully optimized structures of Pd/Si-NW of diameter 1.4~1.5Å. Again, (d) side view, (e) top view and (f) front view of the fully optimized structures of Pd/Si-NW of diameter 2.2~2.3Å.


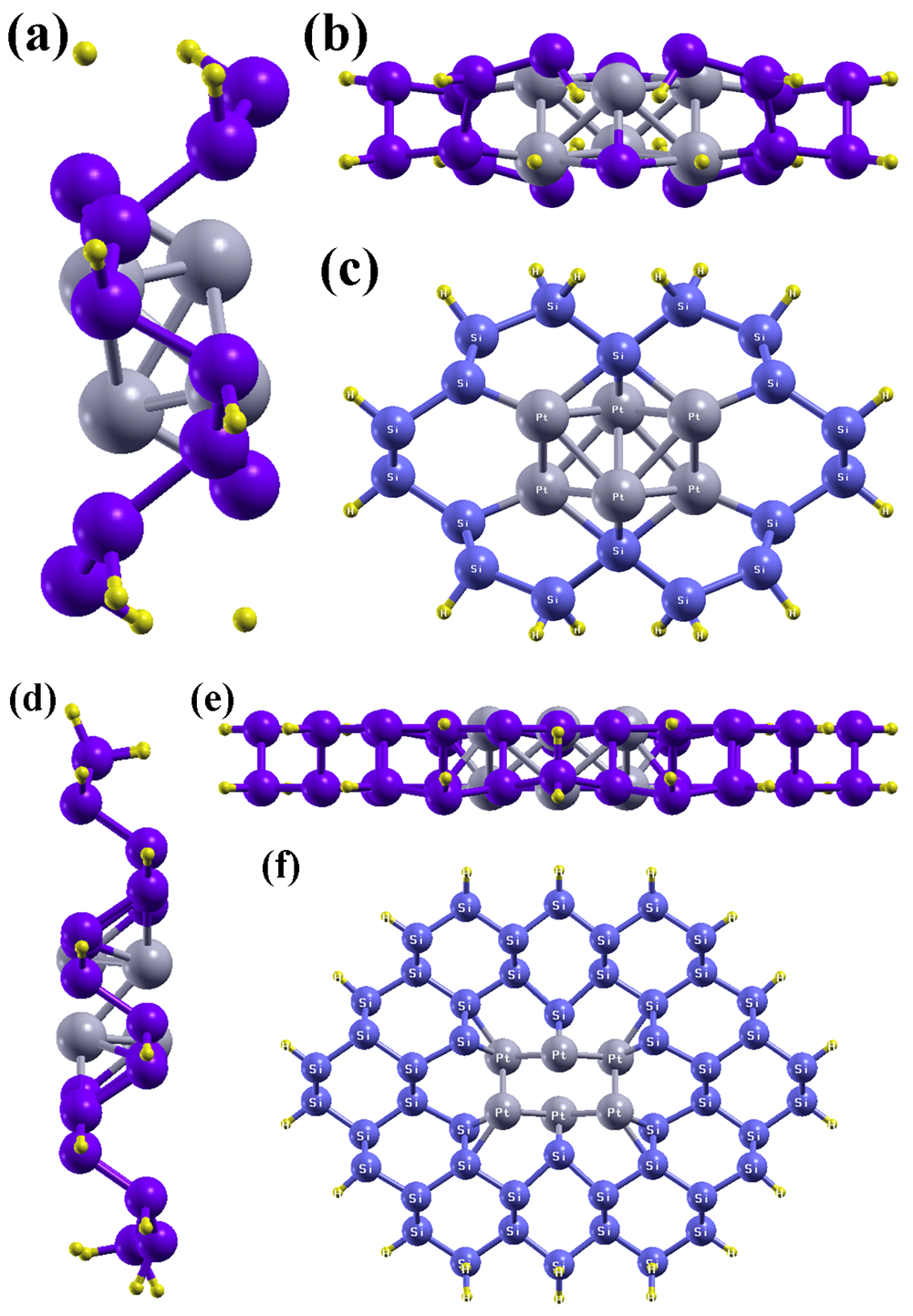


**Figure S11:** (a) Side view, (b) top view and (c) front view of the fully optimized structures of Pt/Si-NW of diameter 1.4~1.5Å. Again, (d) side view, (e) top view and (f) front view of the fully optimized structures of Pt/Si-NW of diameter 2.2~2.3Å.
